# Supplementary material for: Pyruvate Dehydrogenase Kinase 1 inhibition mediated oxidative phosphorylation enhancement in cartilage promotes osteoarthritis progression
Source: BMC Musculoskelet Disord. 2023 Jul 20;24:597. doi: 10.1186/s12891-023-06585-6 (PMC10357736; doi:10.1186/s12891-023-06585-6)
Supplement: Supplementary file 1 — Additional file 1. [file 12891_2023_6585_MOESM1_ESM.zip › MF.Gsea.1653627316242/GOMF_CADHERIN_BINDING.html]

Details for gene set GOMF\_CADHERIN\_BINDING[GSEA]

|  || Dataset | 55457\_collapsed\_to\_symbols.cls.cls #OA\_versus\_normal.cls.cls #OA\_versus\_normal\_repos |
| Phenotype | cls.cls#OA\_versus\_normal\_repos |
| Upregulated in class | normal |
| GeneSet | GOMF\_CADHERIN\_BINDING |
| Enrichment Score (ES) | -0.28313893 |
| Normalized Enrichment Score (NES) | -1.4312692 |
| Nominal p-value | 0.0041265474 |
| FDR q-value | 0.24259213 |
| FWER p-Value | 1.0 |
Table: GSEA Results Summary

  

Fig 1: Enrichment plot: GOMF\_CADHERIN\_BINDING      
 Profile of the Running ES Score & Positions of GeneSet Members on the Rank Ordered List

  

| SYMBOL | TITLE | RANK IN GENE LIST | RANK METRIC SCORE | RUNNING ES | CORE ENRICHMENT || 1 | SNX2 | sorting nexin 2 [Source:HGNC Symbol;Acc:HGNC:11173] | 43 | 1.007 | 0.0074 | No |
| 2 | GLOD4 | glyoxalase domain containing 4 [Source:HGNC Symbol;Acc:HGNC:14111] | 95 | 0.918 | 0.0133 | No |
| 3 | CHMP2B | charged multivesicular body protein 2B [Source:HGNC Symbol;Acc:HGNC:24537] | 129 | 0.876 | 0.0201 | No |
| 4 | PCBP1 | poly(rC) binding protein 1 [Source:HGNC Symbol;Acc:HGNC:8647] | 197 | 0.823 | 0.0237 | No |
| 5 | NCK1 | NCK adaptor protein 1 [Source:HGNC Symbol;Acc:HGNC:7664] | 222 | 0.804 | 0.0304 | No |
| 6 | CORO1B | coronin 1B [Source:HGNC Symbol;Acc:HGNC:2253] | 279 | 0.766 | 0.0343 | No |
| 7 | PTPRO | protein tyrosine phosphatase receptor type O [Source:HGNC Symbol;Acc:HGNC:9678] | 364 | 0.719 | 0.0353 | No |
| 8 | TMPO | thymopoietin [Source:HGNC Symbol;Acc:HGNC:11875] | 398 | 0.707 | 0.0403 | No |
| 9 | ATIC | 5-aminoimidazole-4-carboxamide ribonucleotide formyltransferase/IMP cyclohydrolase [Source:HGNC Symbol;Acc:HGNC:794] | 401 | 0.706 | 0.0478 | No |
| 10 | PSEN1 | presenilin 1 [Source:HGNC Symbol;Acc:HGNC:9508] | 425 | 0.695 | 0.0535 | No |
| 11 | CHMP5 | charged multivesicular body protein 5 [Source:HGNC Symbol;Acc:HGNC:26942] | 556 | 0.638 | 0.0500 | No |
| 12 | PPP1CA | protein phosphatase 1 catalytic subunit alpha [Source:HGNC Symbol;Acc:HGNC:9281] | 603 | 0.622 | 0.0531 | No |
| 13 | H1-10 | H1.10 linker histone [Source:HGNC Symbol;Acc:HGNC:4722] | 766 | 0.577 | 0.0464 | No |
| 14 | TES | testin LIM domain protein [Source:HGNC Symbol;Acc:HGNC:14620] | 850 | 0.554 | 0.0458 | No |
| 15 | DHX29 | DExH-box helicase 29 [Source:HGNC Symbol;Acc:HGNC:15815] | 1012 | 0.515 | 0.0386 | No |
| 16 | EPS8L2 | EPS8 like 2 [Source:HGNC Symbol;Acc:HGNC:21296] | 1134 | 0.489 | 0.0343 | No |
| 17 | PSMB6 | proteasome 20S subunit beta 6 [Source:HGNC Symbol;Acc:HGNC:9543] | 1188 | 0.479 | 0.0352 | No |
| 18 | SH3GLB1 | "SH3 domain containing GRB2 like, endophilin B1 [Source:HGNC Symbol;Acc:HGNC:10833]" | 1216 | 0.473 | 0.0382 | No |
| 19 | CAPG | "capping actin protein, gelsolin like [Source:HGNC Symbol;Acc:HGNC:1474]" | 1245 | 0.468 | 0.0410 | No |
| 20 | CDH11 | cadherin 11 [Source:HGNC Symbol;Acc:HGNC:1750] | 1285 | 0.460 | 0.0429 | No |
| 21 | SNX5 | sorting nexin 5 [Source:HGNC Symbol;Acc:HGNC:14969] | 1379 | 0.446 | 0.0403 | No |
| 22 | PARK7 | Parkinsonism associated deglycase [Source:HGNC Symbol;Acc:HGNC:16369] | 1406 | 0.442 | 0.0430 | No |
| 23 | CDH9 | cadherin 9 [Source:HGNC Symbol;Acc:HGNC:1768] | 1432 | 0.438 | 0.0457 | No |
| 24 | SEPTIN2 | septin 2 [Source:HGNC Symbol;Acc:HGNC:7729] | 1528 | 0.424 | 0.0428 | No |
| 25 | DDX6 | DEAD-box helicase 6 [Source:HGNC Symbol;Acc:HGNC:2747] | 1687 | 0.401 | 0.0345 | No |
| 26 | OLA1 | Obg like ATPase 1 [Source:HGNC Symbol;Acc:HGNC:28833] | 1791 | 0.385 | 0.0305 | No |
| 27 | P2RX4 | purinergic receptor P2X 4 [Source:HGNC Symbol;Acc:HGNC:8535] | 1827 | 0.381 | 0.0318 | No |
| 28 | PFN1 | profilin 1 [Source:HGNC Symbol;Acc:HGNC:8881] | 1863 | 0.377 | 0.0331 | No |
| 29 | TXNDC9 | thioredoxin domain containing 9 [Source:HGNC Symbol;Acc:HGNC:24110] | 1893 | 0.373 | 0.0349 | No |
| 30 | PPFIBP1 | PPFIA binding protein 1 [Source:HGNC Symbol;Acc:HGNC:9249] | 1896 | 0.373 | 0.0387 | No |
| 31 | CTNNA3 | catenin alpha 3 [Source:HGNC Symbol;Acc:HGNC:2511] | 2030 | 0.358 | 0.0320 | No |
| 32 | CDH20 | cadherin 20 [Source:HGNC Symbol;Acc:HGNC:1760] | 2066 | 0.354 | 0.0331 | No |
| 33 | CRKL | "CRK like proto-oncogene, adaptor protein [Source:HGNC Symbol;Acc:HGNC:2363]" | 2283 | 0.329 | 0.0195 | No |
| 34 | H3C10 | H3 clustered histone 10 [Source:HGNC Symbol;Acc:HGNC:4775] | 2287 | 0.328 | 0.0228 | No |
| 35 | EHD4 | EH domain containing 4 [Source:HGNC Symbol;Acc:HGNC:3245] | 2313 | 0.325 | 0.0243 | No |
| 36 | FXYD5 | FXYD domain containing ion transport regulator 5 [Source:HGNC Symbol;Acc:HGNC:4029] | 2405 | 0.314 | 0.0204 | No |
| 37 | LASP1 | LIM and SH3 protein 1 [Source:HGNC Symbol;Acc:HGNC:6513] | 2493 | 0.303 | 0.0168 | No |
| 38 | DBN1 | drebrin 1 [Source:HGNC Symbol;Acc:HGNC:2695] | 2526 | 0.300 | 0.0175 | No |
| 39 | CDH1 | cadherin 1 [Source:HGNC Symbol;Acc:HGNC:1748] | 2589 | 0.292 | 0.0157 | No |
| 40 | EVPL | envoplakin [Source:HGNC Symbol;Acc:HGNC:3503] | 2645 | 0.286 | 0.0144 | No |
| 41 | S100A11 | S100 calcium binding protein A11 [Source:HGNC Symbol;Acc:HGNC:10488] | 2676 | 0.283 | 0.0151 | No |
| 42 | CDH2 | cadherin 2 [Source:HGNC Symbol;Acc:HGNC:1759] | 2679 | 0.282 | 0.0180 | No |
| 43 | CCS | copper chaperone for superoxide dismutase [Source:HGNC Symbol;Acc:HGNC:1613] | 2727 | 0.278 | 0.0172 | No |
| 44 | SNX1 | sorting nexin 1 [Source:HGNC Symbol;Acc:HGNC:11172] | 2906 | 0.260 | 0.0059 | No |
| 45 | GOLGA3 | golgin A3 [Source:HGNC Symbol;Acc:HGNC:4426] | 2922 | 0.258 | 0.0075 | No |
| 46 | WASF2 | WASP family member 2 [Source:HGNC Symbol;Acc:HGNC:12733] | 3101 | 0.239 | -0.0040 | No |
| 47 | PRDX1 | peroxiredoxin 1 [Source:HGNC Symbol;Acc:HGNC:9352] | 3113 | 0.239 | -0.0023 | No |
| 48 | PTPRT | protein tyrosine phosphatase receptor type T [Source:HGNC Symbol;Acc:HGNC:9682] | 3157 | 0.234 | -0.0032 | No |
| 49 | RANBP1 | RAN binding protein 1 [Source:HGNC Symbol;Acc:HGNC:9847] | 3214 | 0.229 | -0.0052 | No |
| 50 | PUF60 | poly(U) binding splicing factor 60 [Source:HGNC Symbol;Acc:HGNC:17042] | 3237 | 0.227 | -0.0045 | No |
| 51 | SEPTIN7 | septin 7 [Source:HGNC Symbol;Acc:HGNC:1717] | 3396 | 0.212 | -0.0148 | No |
| 52 | EMD | emerin [Source:HGNC Symbol;Acc:HGNC:3331] | 3404 | 0.211 | -0.0131 | No |
| 53 | PARVA | parvin alpha [Source:HGNC Symbol;Acc:HGNC:14652] | 3499 | 0.202 | -0.0183 | No |
| 54 | CNN3 | calponin 3 [Source:HGNC Symbol;Acc:HGNC:2157] | 3539 | 0.199 | -0.0193 | No |
| 55 | OLFM4 | olfactomedin 4 [Source:HGNC Symbol;Acc:HGNC:17190] | 3543 | 0.198 | -0.0174 | No |
| 56 | EXOC3 | exocyst complex component 3 [Source:HGNC Symbol;Acc:HGNC:30378] | 3583 | 0.195 | -0.0184 | No |
| 57 | CDH18 | cadherin 18 [Source:HGNC Symbol;Acc:HGNC:1757] | 3659 | 0.189 | -0.0223 | No |
| 58 | TWF2 | twinfilin actin binding protein 2 [Source:HGNC Symbol;Acc:HGNC:9621] | 3765 | 0.180 | -0.0287 | No |
| 59 | EZR | ezrin [Source:HGNC Symbol;Acc:HGNC:12691] | 3817 | 0.174 | -0.0309 | No |
| 60 | LIMA1 | LIM domain and actin binding 1 [Source:HGNC Symbol;Acc:HGNC:24636] | 3867 | 0.168 | -0.0329 | No |
| 61 | ADD1 | adducin 1 [Source:HGNC Symbol;Acc:HGNC:243] | 3872 | 0.167 | -0.0315 | No |
| 62 | SND1 | staphylococcal nuclease and tudor domain containing 1 [Source:HGNC Symbol;Acc:HGNC:30646] | 3897 | 0.165 | -0.0316 | No |
| 63 | ACVR1 | activin A receptor type 1 [Source:HGNC Symbol;Acc:HGNC:171] | 3898 | 0.165 | -0.0298 | No |
| 64 | ABI1 | abl interactor 1 [Source:HGNC Symbol;Acc:HGNC:11320] | 3902 | 0.164 | -0.0283 | No |
| 65 | VAPB | VAMP associated protein B and C [Source:HGNC Symbol;Acc:HGNC:12649] | 3905 | 0.164 | -0.0267 | No |
| 66 | ANK3 | ankyrin 3 [Source:HGNC Symbol;Acc:HGNC:494] | 3921 | 0.163 | -0.0261 | No |
| 67 | PCMT1 | protein-L-isoaspartate (D-aspartate) O-methyltransferase [Source:HGNC Symbol;Acc:HGNC:8728] | 3925 | 0.162 | -0.0246 | No |
| 68 | ZC3HAV1 | "zinc finger CCCH-type containing, antiviral 1 [Source:HGNC Symbol;Acc:HGNC:23721]" | 3946 | 0.161 | -0.0244 | No |
| 69 | CDH19 | cadherin 19 [Source:HGNC Symbol;Acc:HGNC:1758] | 3961 | 0.159 | -0.0238 | No |
| 70 | PKM | pyruvate kinase M1/2 [Source:HGNC Symbol;Acc:HGNC:9021] | 3963 | 0.159 | -0.0222 | No |
| 71 | PPL | periplakin [Source:HGNC Symbol;Acc:HGNC:9273] | 3999 | 0.156 | -0.0233 | No |
| 72 | CTNNA1 | catenin alpha 1 [Source:HGNC Symbol;Acc:HGNC:2509] | 4003 | 0.156 | -0.0219 | No |
| 73 | CTNND1 | catenin delta 1 [Source:HGNC Symbol;Acc:HGNC:2515] | 4243 | 0.133 | -0.0394 | No |
| 74 | KLC2 | kinesin light chain 2 [Source:HGNC Symbol;Acc:HGNC:20716] | 4244 | 0.133 | -0.0380 | No |
| 75 | ALDOA | "aldolase, fructose-bisphosphate A [Source:HGNC Symbol;Acc:HGNC:414]" | 4289 | 0.129 | -0.0401 | No |
| 76 | STXBP6 | syntaxin binding protein 6 [Source:HGNC Symbol;Acc:HGNC:19666] | 4322 | 0.126 | -0.0412 | No |
| 77 | MYO1B | myosin IB [Source:HGNC Symbol;Acc:HGNC:7596] | 4425 | 0.119 | -0.0481 | No |
| 78 | STAT1 | signal transducer and activator of transcription 1 [Source:HGNC Symbol;Acc:HGNC:11362] | 4495 | 0.113 | -0.0523 | No |
| 79 | CSNK1D | casein kinase 1 delta [Source:HGNC Symbol;Acc:HGNC:2452] | 4529 | 0.111 | -0.0537 | No |
| 80 | NUDC | "nuclear distribution C, dynein complex regulator [Source:HGNC Symbol;Acc:HGNC:8045]" | 4536 | 0.110 | -0.0530 | No |
| 81 | CD46 | CD46 molecule [Source:HGNC Symbol;Acc:HGNC:6953] | 4563 | 0.108 | -0.0539 | No |
| 82 | CDH22 | cadherin 22 [Source:HGNC Symbol;Acc:HGNC:13251] | 4586 | 0.106 | -0.0545 | No |
| 83 | ARVCF | ARVCF delta catenin family member [Source:HGNC Symbol;Acc:HGNC:728] | 4591 | 0.105 | -0.0537 | No |
| 84 | CDH12 | cadherin 12 [Source:HGNC Symbol;Acc:HGNC:1751] | 4632 | 0.101 | -0.0558 | No |
| 85 | PLIN3 | perilipin 3 [Source:HGNC Symbol;Acc:HGNC:16893] | 4692 | 0.097 | -0.0594 | No |
| 86 | CLIC1 | chloride intracellular channel 1 [Source:HGNC Symbol;Acc:HGNC:2062] | 4704 | 0.095 | -0.0593 | No |
| 87 | HCFC1 | host cell factor C1 [Source:HGNC Symbol;Acc:HGNC:4839] | 4937 | 0.077 | -0.0769 | No |
| 88 | USO1 | USO1 vesicle transport factor [Source:HGNC Symbol;Acc:HGNC:30904] | 4992 | 0.074 | -0.0804 | No |
| 89 | CAPZB | capping actin protein of muscle Z-line subunit beta [Source:HGNC Symbol;Acc:HGNC:1491] | 5014 | 0.072 | -0.0813 | No |
| 90 | MAPRE1 | microtubule associated protein RP/EB family member 1 [Source:HGNC Symbol;Acc:HGNC:6890] | 5117 | 0.063 | -0.0887 | No |
| 91 | EIF4H | eukaryotic translation initiation factor 4H [Source:HGNC Symbol;Acc:HGNC:12741] | 5118 | 0.062 | -0.0880 | No |
| 92 | CDH4 | cadherin 4 [Source:HGNC Symbol;Acc:HGNC:1763] | 5121 | 0.062 | -0.0875 | No |
| 93 | SPTAN1 | "spectrin alpha, non-erythrocytic 1 [Source:HGNC Symbol;Acc:HGNC:11273]" | 5131 | 0.062 | -0.0876 | No |
| 94 | VASP | vasodilator stimulated phosphoprotein [Source:HGNC Symbol;Acc:HGNC:12652] | 5143 | 0.060 | -0.0878 | No |
| 95 | SPTBN2 | "spectrin beta, non-erythrocytic 2 [Source:HGNC Symbol;Acc:HGNC:11276]" | 5200 | 0.056 | -0.0916 | No |
| 96 | PKP1 | plakophilin 1 [Source:HGNC Symbol;Acc:HGNC:9023] | 5248 | 0.052 | -0.0948 | No |
| 97 | CLINT1 | clathrin interactor 1 [Source:HGNC Symbol;Acc:HGNC:23186] | 5253 | 0.052 | -0.0945 | No |
| 98 | EFHD2 | EF-hand domain family member D2 [Source:HGNC Symbol;Acc:HGNC:28670] | 5276 | 0.050 | -0.0958 | No |
| 99 | RPS26 | ribosomal protein S26 [Source:HGNC Symbol;Acc:HGNC:10414] | 5368 | 0.043 | -0.1025 | No |
| 100 | IST1 | IST1 factor associated with ESCRT-III [Source:HGNC Symbol;Acc:HGNC:28977] | 5370 | 0.043 | -0.1021 | No |
| 101 | RARS1 | arginyl-tRNA synthetase 1 [Source:HGNC Symbol;Acc:HGNC:9870] | 5469 | 0.035 | -0.1095 | No |
| 102 | PFKP | "phosphofructokinase, platelet [Source:HGNC Symbol;Acc:HGNC:8878]" | 5500 | 0.032 | -0.1116 | No |
| 103 | PKP3 | plakophilin 3 [Source:HGNC Symbol;Acc:HGNC:9025] | 5510 | 0.032 | -0.1119 | No |
| 104 | CNN2 | calponin 2 [Source:HGNC Symbol;Acc:HGNC:2156] | 5533 | 0.030 | -0.1134 | No |
| 105 | H3C7 | H3 clustered histone 7 [Source:HGNC Symbol;Acc:HGNC:4773] | 5716 | 0.014 | -0.1277 | No |
| 106 | PROM1 | prominin 1 [Source:HGNC Symbol;Acc:HGNC:9454] | 5720 | 0.014 | -0.1278 | No |
| 107 | EPCAM | epithelial cell adhesion molecule [Source:HGNC Symbol;Acc:HGNC:11529] | 5769 | 0.010 | -0.1315 | No |
| 108 | YWHAZ | tyrosine 3-monooxygenase/tryptophan 5-monooxygenase activation protein zeta [Source:HGNC Symbol;Acc:HGNC:12855] | 5770 | 0.010 | -0.1313 | No |
| 109 | YKT6 | YKT6 v-SNARE homolog [Source:HGNC Symbol;Acc:HGNC:16959] | 5800 | 0.008 | -0.1336 | No |
| 110 | HDLBP | high density lipoprotein binding protein [Source:HGNC Symbol;Acc:HGNC:4857] | 5879 | 0.004 | -0.1397 | No |
| 111 | ABCF3 | ATP binding cassette subfamily F member 3 [Source:HGNC Symbol;Acc:HGNC:72] | 5904 | 0.002 | -0.1416 | No |
| 112 | CAST | calpastatin [Source:HGNC Symbol;Acc:HGNC:1515] | 5941 | -0.001 | -0.1445 | No |
| 113 | TMOD3 | tropomodulin 3 [Source:HGNC Symbol;Acc:HGNC:11873] | 5944 | -0.001 | -0.1446 | No |
| 114 | RACK1 | receptor for activated C kinase 1 [Source:HGNC Symbol;Acc:HGNC:4399] | 5983 | -0.003 | -0.1476 | No |
| 115 | GIGYF2 | GRB10 interacting GYF protein 2 [Source:HGNC Symbol;Acc:HGNC:11960] | 6123 | -0.014 | -0.1585 | No |
| 116 | ERC1 | ELKS/RAB6-interacting/CAST family member 1 [Source:HGNC Symbol;Acc:HGNC:17072] | 6147 | -0.016 | -0.1601 | No |
| 117 | RPL15 | ribosomal protein L15 [Source:HGNC Symbol;Acc:HGNC:10306] | 6185 | -0.019 | -0.1629 | No |
| 118 | DLG1 | discs large MAGUK scaffold protein 1 [Source:HGNC Symbol;Acc:HGNC:2900] | 6219 | -0.022 | -0.1652 | No |
| 119 | CKAP5 | cytoskeleton associated protein 5 [Source:HGNC Symbol;Acc:HGNC:28959] | 6237 | -0.023 | -0.1663 | No |
| 120 | H3C3 | H3 clustered histone 3 [Source:HGNC Symbol;Acc:HGNC:4768] | 6308 | -0.028 | -0.1716 | No |
| 121 | FLNB | filamin B [Source:HGNC Symbol;Acc:HGNC:3755] | 6499 | -0.042 | -0.1862 | No |
| 122 | CCT8 | chaperonin containing TCP1 subunit 8 [Source:HGNC Symbol;Acc:HGNC:1623] | 6529 | -0.044 | -0.1880 | No |
| 123 | CC2D1A | coiled-coil and C2 domain containing 1A [Source:HGNC Symbol;Acc:HGNC:30237] | 6541 | -0.045 | -0.1884 | No |
| 124 | H3C6 | H3 clustered histone 6 [Source:HGNC Symbol;Acc:HGNC:4769] | 6782 | -0.061 | -0.2068 | No |
| 125 | PRDX6 | peroxiredoxin 6 [Source:HGNC Symbol;Acc:HGNC:16753] | 6783 | -0.061 | -0.2062 | No |
| 126 | EHD1 | EH domain containing 1 [Source:HGNC Symbol;Acc:HGNC:3242] | 6846 | -0.067 | -0.2104 | No |
| 127 | EIF2S3 | eukaryotic translation initiation factor 2 subunit gamma [Source:HGNC Symbol;Acc:HGNC:3267] | 6870 | -0.069 | -0.2115 | No |
| 128 | EEF1D | eukaryotic translation elongation factor 1 delta [Source:HGNC Symbol;Acc:HGNC:3211] | 6923 | -0.074 | -0.2148 | No |
| 129 | STX5 | syntaxin 5 [Source:HGNC Symbol;Acc:HGNC:11440] | 6932 | -0.074 | -0.2146 | No |
| 130 | EIF5 | eukaryotic translation initiation factor 5 [Source:HGNC Symbol;Acc:HGNC:3299] | 6934 | -0.074 | -0.2139 | No |
| 131 | SRC | "SRC proto-oncogene, non-receptor tyrosine kinase [Source:HGNC Symbol;Acc:HGNC:11283]" | 6940 | -0.075 | -0.2135 | No |
| 132 | KRT18 | keratin 18 [Source:HGNC Symbol;Acc:HGNC:6430] | 7027 | -0.082 | -0.2194 | No |
| 133 | CDH10 | cadherin 10 [Source:HGNC Symbol;Acc:HGNC:1749] | 7032 | -0.082 | -0.2189 | No |
| 134 | AFDN | "afadin, adherens junction formation factor [Source:HGNC Symbol;Acc:HGNC:7137]" | 7077 | -0.086 | -0.2214 | No |
| 135 | EPS8L1 | EPS8 like 1 [Source:HGNC Symbol;Acc:HGNC:21295] | 7083 | -0.086 | -0.2209 | No |
| 136 | IDH1 | isocitrate dehydrogenase (NADP(+)) 1 [Source:HGNC Symbol;Acc:HGNC:5382] | 7109 | -0.089 | -0.2219 | No |
| 137 | PLCB3 | phospholipase C beta 3 [Source:HGNC Symbol;Acc:HGNC:9056] | 7118 | -0.090 | -0.2216 | No |
| 138 | NUMB | NUMB endocytic adaptor protein [Source:HGNC Symbol;Acc:HGNC:8060] | 7180 | -0.095 | -0.2254 | No |
| 139 | RAB11B | "RAB11B, member RAS oncogene family [Source:HGNC Symbol;Acc:HGNC:9761]" | 7227 | -0.097 | -0.2280 | No |
| 140 | TWF1 | twinfilin actin binding protein 1 [Source:HGNC Symbol;Acc:HGNC:9620] | 7232 | -0.098 | -0.2273 | No |
| 141 | CD2AP | CD2 associated protein [Source:HGNC Symbol;Acc:HGNC:14258] | 7262 | -0.100 | -0.2285 | No |
| 142 | CALD1 | caldesmon 1 [Source:HGNC Symbol;Acc:HGNC:1441] | 7359 | -0.107 | -0.2350 | No |
| 143 | H3C12 | H3 clustered histone 12 [Source:HGNC Symbol;Acc:HGNC:4774] | 7371 | -0.108 | -0.2347 | No |
| 144 | PDXDC1 | pyridoxal dependent decarboxylase domain containing 1 [Source:HGNC Symbol;Acc:HGNC:28995] | 7373 | -0.108 | -0.2336 | No |
| 145 | LRRC59 | leucine rich repeat containing 59 [Source:HGNC Symbol;Acc:HGNC:28817] | 7389 | -0.109 | -0.2336 | No |
| 146 | PI4KA | phosphatidylinositol 4-kinase alpha [Source:HGNC Symbol;Acc:HGNC:8983] | 7397 | -0.110 | -0.2330 | No |
| 147 | TBC1D2 | TBC1 domain family member 2 [Source:HGNC Symbol;Acc:HGNC:18026] | 7399 | -0.111 | -0.2319 | No |
| 148 | DCHS1 | dachsous cadherin-related 1 [Source:HGNC Symbol;Acc:HGNC:13681] | 7464 | -0.115 | -0.2357 | No |
| 149 | RUVBL1 | RuvB like AAA ATPase 1 [Source:HGNC Symbol;Acc:HGNC:10474] | 7477 | -0.116 | -0.2354 | No |
| 150 | PPME1 | protein phosphatase methylesterase 1 [Source:HGNC Symbol;Acc:HGNC:30178] | 7483 | -0.117 | -0.2345 | No |
| 151 | COBLL1 | cordon-bleu WH2 repeat protein like 1 [Source:HGNC Symbol;Acc:HGNC:23571] | 7561 | -0.124 | -0.2393 | No |
| 152 | VCL | vinculin [Source:HGNC Symbol;Acc:HGNC:12665] | 7687 | -0.133 | -0.2478 | No |
| 153 | GIPC1 | GIPC PDZ domain containing family member 1 [Source:HGNC Symbol;Acc:HGNC:1226] | 7712 | -0.135 | -0.2483 | No |
| 154 | PKP2 | plakophilin 2 [Source:HGNC Symbol;Acc:HGNC:9024] | 7731 | -0.136 | -0.2482 | No |
| 155 | PAK2 | p21 (RAC1) activated kinase 2 [Source:HGNC Symbol;Acc:HGNC:8591] | 7762 | -0.139 | -0.2491 | No |
| 156 | MMP24 | matrix metallopeptidase 24 [Source:HGNC Symbol;Acc:HGNC:7172] | 7767 | -0.139 | -0.2479 | No |
| 157 | CTNND2 | catenin delta 2 [Source:HGNC Symbol;Acc:HGNC:2516] | 7851 | -0.144 | -0.2530 | No |
| 158 | HSPA1A | heat shock protein family A (Hsp70) member 1A [Source:HGNC Symbol;Acc:HGNC:5232] | 7876 | -0.146 | -0.2533 | No |
| 159 | NOTCH3 | notch receptor 3 [Source:HGNC Symbol;Acc:HGNC:7883] | 7894 | -0.147 | -0.2530 | No |
| 160 | CDH3 | cadherin 3 [Source:HGNC Symbol;Acc:HGNC:1762] | 7924 | -0.149 | -0.2537 | No |
| 161 | PPP1R13L | protein phosphatase 1 regulatory subunit 13 like [Source:HGNC Symbol;Acc:HGNC:18838] | 7967 | -0.153 | -0.2554 | No |
| 162 | USP8 | ubiquitin specific peptidase 8 [Source:HGNC Symbol;Acc:HGNC:12631] | 8031 | -0.158 | -0.2587 | No |
| 163 | PTPRB | protein tyrosine phosphatase receptor type B [Source:HGNC Symbol;Acc:HGNC:9665] | 8044 | -0.158 | -0.2580 | No |
| 164 | SFN | stratifin [Source:HGNC Symbol;Acc:HGNC:10773] | 8118 | -0.164 | -0.2620 | No |
| 165 | BZW2 | basic leucine zipper and W2 domains 2 [Source:HGNC Symbol;Acc:HGNC:18808] | 8132 | -0.165 | -0.2613 | No |
| 166 | CDH6 | cadherin 6 [Source:HGNC Symbol;Acc:HGNC:1765] | 8135 | -0.165 | -0.2596 | No |
| 167 | H3C1 | H3 clustered histone 1 [Source:HGNC Symbol;Acc:HGNC:4766] | 8270 | -0.177 | -0.2684 | No |
| 168 | IQGAP1 | IQ motif containing GTPase activating protein 1 [Source:HGNC Symbol;Acc:HGNC:6110] | 8276 | -0.177 | -0.2669 | No |
| 169 | RSL1D1 | ribosomal L1 domain containing 1 [Source:HGNC Symbol;Acc:HGNC:24534] | 8286 | -0.177 | -0.2657 | No |
| 170 | CTNNAL1 | catenin alpha like 1 [Source:HGNC Symbol;Acc:HGNC:2512] | 8288 | -0.178 | -0.2638 | No |
| 171 | SPTBN1 | "spectrin beta, non-erythrocytic 1 [Source:HGNC Symbol;Acc:HGNC:11275]" | 8335 | -0.181 | -0.2655 | No |
| 172 | SH3GL1 | "SH3 domain containing GRB2 like 1, endophilin A2 [Source:HGNC Symbol;Acc:HGNC:10830]" | 8440 | -0.189 | -0.2717 | No |
| 173 | MYH9 | myosin heavy chain 9 [Source:HGNC Symbol;Acc:HGNC:7579] | 8463 | -0.191 | -0.2714 | No |
| 174 | FSCN1 | fascin actin-bundling protein 1 [Source:HGNC Symbol;Acc:HGNC:11148] | 8498 | -0.194 | -0.2720 | No |
| 175 | TJP1 | tight junction protein 1 [Source:HGNC Symbol;Acc:HGNC:11827] | 8523 | -0.195 | -0.2718 | No |
| 176 | TRIM29 | tripartite motif containing 29 [Source:HGNC Symbol;Acc:HGNC:17274] | 8594 | -0.201 | -0.2752 | No |
| 177 | RPL29 | ribosomal protein L29 [Source:HGNC Symbol;Acc:HGNC:10331] | 8661 | -0.205 | -0.2783 | No |
| 178 | NDRG1 | N-myc downstream regulated 1 [Source:HGNC Symbol;Acc:HGNC:7679] | 8688 | -0.208 | -0.2781 | No |
| 179 | AHSA1 | activator of HSP90 ATPase activity 1 [Source:HGNC Symbol;Acc:HGNC:1189] | 8706 | -0.210 | -0.2772 | No |
| 180 | PACSIN2 | protein kinase C and casein kinase substrate in neurons 2 [Source:HGNC Symbol;Acc:HGNC:8571] | 8727 | -0.211 | -0.2765 | No |
| 181 | DIAPH3 | diaphanous related formin 3 [Source:HGNC Symbol;Acc:HGNC:15480] | 8758 | -0.213 | -0.2766 | No |
| 182 | PTPN1 | protein tyrosine phosphatase non-receptor type 1 [Source:HGNC Symbol;Acc:HGNC:9642] | 8829 | -0.220 | -0.2798 | No |
| 183 | SLC9A3R2 | SLC9A3 regulator 2 [Source:HGNC Symbol;Acc:HGNC:11076] | 8831 | -0.220 | -0.2775 | No |
| 184 | VAPA | VAMP associated protein A [Source:HGNC Symbol;Acc:HGNC:12648] | 8854 | -0.221 | -0.2768 | No |
| 185 | PICALM | phosphatidylinositol binding clathrin assembly protein [Source:HGNC Symbol;Acc:HGNC:15514] | 8905 | -0.225 | -0.2784 | No |
| 186 | SHTN1 | shootin 1 [Source:HGNC Symbol;Acc:HGNC:29319] | 8966 | -0.229 | -0.2807 | Yes |
| 187 | H3C2 | H3 clustered histone 2 [Source:HGNC Symbol;Acc:HGNC:4776] | 8970 | -0.230 | -0.2784 | Yes |
| 188 | ARFIP2 | ADP ribosylation factor interacting protein 2 [Source:HGNC Symbol;Acc:HGNC:17160] | 8976 | -0.230 | -0.2763 | Yes |
| 189 | BSG | basigin (Ok blood group) [Source:HGNC Symbol;Acc:HGNC:1116] | 9043 | -0.235 | -0.2790 | Yes |
| 190 | PAK6 | p21 (RAC1) activated kinase 6 [Source:HGNC Symbol;Acc:HGNC:16061] | 9068 | -0.237 | -0.2784 | Yes |
| 191 | MPRIP | myosin phosphatase Rho interacting protein [Source:HGNC Symbol;Acc:HGNC:30321] | 9093 | -0.239 | -0.2777 | Yes |
| 192 | ITGA6 | integrin subunit alpha 6 [Source:HGNC Symbol;Acc:HGNC:6142] | 9112 | -0.240 | -0.2766 | Yes |
| 193 | MACF1 | microtubule actin crosslinking factor 1 [Source:HGNC Symbol;Acc:HGNC:13664] | 9123 | -0.241 | -0.2748 | Yes |
| 194 | TLN1 | talin 1 [Source:HGNC Symbol;Acc:HGNC:11845] | 9131 | -0.242 | -0.2727 | Yes |
| 195 | HSPA8 | heat shock protein family A (Hsp70) member 8 [Source:HGNC Symbol;Acc:HGNC:5241] | 9140 | -0.243 | -0.2707 | Yes |
| 196 | PDLIM1 | PDZ and LIM domain 1 [Source:HGNC Symbol;Acc:HGNC:2067] | 9183 | -0.246 | -0.2714 | Yes |
| 197 | SERBP1 | SERPINE1 mRNA binding protein 1 [Source:HGNC Symbol;Acc:HGNC:17860] | 9238 | -0.251 | -0.2730 | Yes |
| 198 | RDX | radixin [Source:HGNC Symbol;Acc:HGNC:9944] | 9239 | -0.251 | -0.2703 | Yes |
| 199 | UNC45A | unc-45 myosin chaperone A [Source:HGNC Symbol;Acc:HGNC:30594] | 9267 | -0.253 | -0.2697 | Yes |
| 200 | ARHGAP1 | Rho GTPase activating protein 1 [Source:HGNC Symbol;Acc:HGNC:673] | 9307 | -0.256 | -0.2700 | Yes |
| 201 | GCN1 | GCN1 activator of EIF2AK4 [Source:HGNC Symbol;Acc:HGNC:4199] | 9327 | -0.258 | -0.2687 | Yes |
| 202 | SEPTIN9 | septin 9 [Source:HGNC Symbol;Acc:HGNC:7323] | 9336 | -0.258 | -0.2666 | Yes |
| 203 | SLK | STE20 like kinase [Source:HGNC Symbol;Acc:HGNC:11088] | 9362 | -0.261 | -0.2658 | Yes |
| 204 | CDH15 | cadherin 15 [Source:HGNC Symbol;Acc:HGNC:1754] | 9379 | -0.262 | -0.2642 | Yes |
| 205 | DNAJB1 | DnaJ heat shock protein family (Hsp40) member B1 [Source:HGNC Symbol;Acc:HGNC:5270] | 9380 | -0.262 | -0.2614 | Yes |
| 206 | EPS15 | epidermal growth factor receptor pathway substrate 15 [Source:HGNC Symbol;Acc:HGNC:3419] | 9409 | -0.265 | -0.2607 | Yes |
| 207 | PTPRJ | protein tyrosine phosphatase receptor type J [Source:HGNC Symbol;Acc:HGNC:9673] | 9463 | -0.270 | -0.2620 | Yes |
| 208 | PAICS | phosphoribosylaminoimidazole carboxylase and phosphoribosylaminoimidazolesuccinocarboxamide synthase [Source:HGNC Symbol;Acc:HGNC:8587] | 9475 | -0.272 | -0.2600 | Yes |
| 209 | RAN | "RAN, member RAS oncogene family [Source:HGNC Symbol;Acc:HGNC:9846]" | 9478 | -0.272 | -0.2572 | Yes |
| 210 | CBL | Cbl proto-oncogene [Source:HGNC Symbol;Acc:HGNC:1541] | 9479 | -0.272 | -0.2543 | Yes |
| 211 | DOCK9 | dedicator of cytokinesis 9 [Source:HGNC Symbol;Acc:HGNC:14132] | 9545 | -0.278 | -0.2564 | Yes |
| 212 | RAB1A | "RAB1A, member RAS oncogene family [Source:HGNC Symbol;Acc:HGNC:9758]" | 9604 | -0.284 | -0.2580 | Yes |
| 213 | ENO1 | enolase 1 [Source:HGNC Symbol;Acc:HGNC:3350] | 9606 | -0.284 | -0.2550 | Yes |
| 214 | MARK2 | microtubule affinity regulating kinase 2 [Source:HGNC Symbol;Acc:HGNC:3332] | 9662 | -0.288 | -0.2563 | Yes |
| 215 | F11R | F11 receptor [Source:HGNC Symbol;Acc:HGNC:14685] | 9765 | -0.297 | -0.2612 | Yes |
| 216 | TJP2 | tight junction protein 2 [Source:HGNC Symbol;Acc:HGNC:11828] | 9772 | -0.298 | -0.2584 | Yes |
| 217 | H3C11 | H3 clustered histone 11 [Source:HGNC Symbol;Acc:HGNC:4771] | 9808 | -0.302 | -0.2579 | Yes |
| 218 | BAIAP2 | BAR/IMD domain containing adaptor protein 2 [Source:HGNC Symbol;Acc:HGNC:947] | 9892 | -0.311 | -0.2612 | Yes |
| 219 | STK38 | serine/threonine kinase 38 [Source:HGNC Symbol;Acc:HGNC:17847] | 9912 | -0.313 | -0.2593 | Yes |
| 220 | CDH5 | cadherin 5 [Source:HGNC Symbol;Acc:HGNC:1764] | 9946 | -0.316 | -0.2585 | Yes |
| 221 | FLNA | filamin A [Source:HGNC Symbol;Acc:HGNC:3754] | 9976 | -0.318 | -0.2574 | Yes |
| 222 | PAK4 | p21 (RAC1) activated kinase 4 [Source:HGNC Symbol;Acc:HGNC:16059] | 10004 | -0.322 | -0.2561 | Yes |
| 223 | SWAP70 | switching B cell complex subunit SWAP70 [Source:HGNC Symbol;Acc:HGNC:17070] | 10019 | -0.323 | -0.2537 | Yes |
| 224 | S100P | S100 calcium binding protein P [Source:HGNC Symbol;Acc:HGNC:10504] | 10050 | -0.326 | -0.2526 | Yes |
| 225 | HSP90AB1 | heat shock protein 90 alpha family class B member 1 [Source:HGNC Symbol;Acc:HGNC:5258] | 10060 | -0.327 | -0.2498 | Yes |
| 226 | FNBP1L | formin binding protein 1 like [Source:HGNC Symbol;Acc:HGNC:20851] | 10082 | -0.329 | -0.2479 | Yes |
| 227 | JUP | junction plakoglobin [Source:HGNC Symbol;Acc:HGNC:6207] | 10101 | -0.331 | -0.2457 | Yes |
| 228 | CDC42EP1 | CDC42 effector protein 1 [Source:HGNC Symbol;Acc:HGNC:17014] | 10147 | -0.336 | -0.2457 | Yes |
| 229 | PDLIM5 | PDZ and LIM domain 5 [Source:HGNC Symbol;Acc:HGNC:17468] | 10158 | -0.337 | -0.2429 | Yes |
| 230 | EPHA2 | EPH receptor A2 [Source:HGNC Symbol;Acc:HGNC:3386] | 10219 | -0.343 | -0.2439 | Yes |
| 231 | H3C4 | H3 clustered histone 4 [Source:HGNC Symbol;Acc:HGNC:4767] | 10258 | -0.347 | -0.2432 | Yes |
| 232 | RPL24 | ribosomal protein L24 [Source:HGNC Symbol;Acc:HGNC:10325] | 10263 | -0.347 | -0.2398 | Yes |
| 233 | ASAP1 | "ArfGAP with SH3 domain, ankyrin repeat and PH domain 1 [Source:HGNC Symbol;Acc:HGNC:2720]" | 10276 | -0.349 | -0.2370 | Yes |
| 234 | ARHGEF16 | Rho guanine nucleotide exchange factor 16 [Source:HGNC Symbol;Acc:HGNC:15515] | 10278 | -0.349 | -0.2333 | Yes |
| 235 | GOLGA2 | golgin A2 [Source:HGNC Symbol;Acc:HGNC:4425] | 10285 | -0.349 | -0.2300 | Yes |
| 236 | RANGAP1 | Ran GTPase activating protein 1 [Source:HGNC Symbol;Acc:HGNC:9854] | 10288 | -0.349 | -0.2264 | Yes |
| 237 | TRPC4 | transient receptor potential cation channel subfamily C member 4 [Source:HGNC Symbol;Acc:HGNC:12336] | 10329 | -0.354 | -0.2257 | Yes |
| 238 | EPS15L1 | epidermal growth factor receptor pathway substrate 15 like 1 [Source:HGNC Symbol;Acc:HGNC:24634] | 10397 | -0.360 | -0.2272 | Yes |
| 239 | GAPVD1 | GTPase activating protein and VPS9 domains 1 [Source:HGNC Symbol;Acc:HGNC:23375] | 10410 | -0.362 | -0.2242 | Yes |
| 240 | APC | APC regulator of WNT signaling pathway [Source:HGNC Symbol;Acc:HGNC:583] | 10440 | -0.366 | -0.2226 | Yes |
| 241 | EPN2 | epsin 2 [Source:HGNC Symbol;Acc:HGNC:18639] | 10446 | -0.367 | -0.2190 | Yes |
| 242 | SCRIB | scribble planar cell polarity protein [Source:HGNC Symbol;Acc:HGNC:30377] | 10496 | -0.372 | -0.2189 | Yes |
| 243 | CDH7 | cadherin 7 [Source:HGNC Symbol;Acc:HGNC:1766] | 10539 | -0.377 | -0.2182 | Yes |
| 244 | SH3GLB2 | "SH3 domain containing GRB2 like, endophilin B2 [Source:HGNC Symbol;Acc:HGNC:10834]" | 10566 | -0.380 | -0.2161 | Yes |
| 245 | CCNB2 | cyclin B2 [Source:HGNC Symbol;Acc:HGNC:1580] | 10639 | -0.387 | -0.2177 | Yes |
| 246 | CDH17 | cadherin 17 [Source:HGNC Symbol;Acc:HGNC:1756] | 10667 | -0.390 | -0.2156 | Yes |
| 247 | PLEC | plectin [Source:HGNC Symbol;Acc:HGNC:9069] | 10799 | -0.405 | -0.2217 | Yes |
| 248 | RPL7A | ribosomal protein L7a [Source:HGNC Symbol;Acc:HGNC:10364] | 10811 | -0.406 | -0.2182 | Yes |
| 249 | CDH8 | cadherin 8 [Source:HGNC Symbol;Acc:HGNC:1767] | 10814 | -0.407 | -0.2139 | Yes |
| 250 | HSPA5 | heat shock protein family A (Hsp70) member 5 [Source:HGNC Symbol;Acc:HGNC:5238] | 10820 | -0.407 | -0.2099 | Yes |
| 251 | ANXA1 | annexin A1 [Source:HGNC Symbol;Acc:HGNC:533] | 10824 | -0.407 | -0.2058 | Yes |
| 252 | LYPLA2 | lysophospholipase 2 [Source:HGNC Symbol;Acc:HGNC:6738] | 10860 | -0.411 | -0.2041 | Yes |
| 253 | AHNAK | AHNAK nucleoprotein [Source:HGNC Symbol;Acc:HGNC:347] | 10903 | -0.417 | -0.2030 | Yes |
| 254 | EIF3E | eukaryotic translation initiation factor 3 subunit E [Source:HGNC Symbol;Acc:HGNC:3277] | 10944 | -0.424 | -0.2016 | Yes |
| 255 | LARP1 | "La ribonucleoprotein 1, translational regulator [Source:HGNC Symbol;Acc:HGNC:29531]" | 10964 | -0.425 | -0.1985 | Yes |
| 256 | CDK5R1 | cyclin dependent kinase 5 regulatory subunit 1 [Source:HGNC Symbol;Acc:HGNC:1775] | 10985 | -0.428 | -0.1955 | Yes |
| 257 | GPRC5A | G protein-coupled receptor class C group 5 member A [Source:HGNC Symbol;Acc:HGNC:9836] | 11112 | -0.445 | -0.2007 | Yes |
| 258 | RPL6 | ribosomal protein L6 [Source:HGNC Symbol;Acc:HGNC:10362] | 11129 | -0.447 | -0.1971 | Yes |
| 259 | LDHA | lactate dehydrogenase A [Source:HGNC Symbol;Acc:HGNC:6535] | 11134 | -0.448 | -0.1926 | Yes |
| 260 | RPL14 | ribosomal protein L14 [Source:HGNC Symbol;Acc:HGNC:10305] | 11154 | -0.451 | -0.1893 | Yes |
| 261 | BMPR2 | bone morphogenetic protein receptor type 2 [Source:HGNC Symbol;Acc:HGNC:1078] | 11174 | -0.454 | -0.1859 | Yes |
| 262 | ZC3H15 | zinc finger CCCH-type containing 15 [Source:HGNC Symbol;Acc:HGNC:29528] | 11243 | -0.463 | -0.1863 | Yes |
| 263 | PTPN11 | protein tyrosine phosphatase non-receptor type 11 [Source:HGNC Symbol;Acc:HGNC:9644] | 11260 | -0.465 | -0.1825 | Yes |
| 264 | PTPRM | protein tyrosine phosphatase receptor type M [Source:HGNC Symbol;Acc:HGNC:9675] | 11289 | -0.470 | -0.1797 | Yes |
| 265 | EIF4G2 | eukaryotic translation initiation factor 4 gamma 2 [Source:HGNC Symbol;Acc:HGNC:3297] | 11313 | -0.473 | -0.1764 | Yes |
| 266 | CTNNA2 | catenin alpha 2 [Source:HGNC Symbol;Acc:HGNC:2510] | 11342 | -0.477 | -0.1735 | Yes |
| 267 | STK24 | serine/threonine kinase 24 [Source:HGNC Symbol;Acc:HGNC:11403] | 11409 | -0.488 | -0.1735 | Yes |
| 268 | KDR | kinase insert domain receptor [Source:HGNC Symbol;Acc:HGNC:6307] | 11454 | -0.496 | -0.1716 | Yes |
| 269 | MRE11 | "MRE11 homolog, double strand break repair nuclease [Source:HGNC Symbol;Acc:HGNC:7230]" | 11458 | -0.497 | -0.1665 | Yes |
| 270 | ANXA2 | annexin A2 [Source:HGNC Symbol;Acc:HGNC:537] | 11472 | -0.498 | -0.1622 | Yes |
| 271 | YWHAB | tyrosine 3-monooxygenase/tryptophan 5-monooxygenase activation protein beta [Source:HGNC Symbol;Acc:HGNC:12849] | 11488 | -0.500 | -0.1580 | Yes |
| 272 | BAG3 | BAG cochaperone 3 [Source:HGNC Symbol;Acc:HGNC:939] | 11562 | -0.509 | -0.1583 | Yes |
| 273 | MRTFB | myocardin related transcription factor B [Source:HGNC Symbol;Acc:HGNC:29819] | 11668 | -0.527 | -0.1609 | Yes |
| 274 | CTTN | cortactin [Source:HGNC Symbol;Acc:HGNC:3338] | 11677 | -0.528 | -0.1559 | Yes |
| 275 | UBFD1 | ubiquitin family domain containing 1 [Source:HGNC Symbol;Acc:HGNC:30565] | 11703 | -0.533 | -0.1521 | Yes |
| 276 | TAGLN2 | transgelin 2 [Source:HGNC Symbol;Acc:HGNC:11554] | 11724 | -0.537 | -0.1479 | Yes |
| 277 | EEF1G | eukaryotic translation elongation factor 1 gamma [Source:HGNC Symbol;Acc:HGNC:3213] | 11767 | -0.544 | -0.1454 | Yes |
| 278 | UBAP2 | ubiquitin associated protein 2 [Source:HGNC Symbol;Acc:HGNC:14185] | 11829 | -0.555 | -0.1442 | Yes |
| 279 | TRIM25 | tripartite motif containing 25 [Source:HGNC Symbol;Acc:HGNC:12932] | 11834 | -0.556 | -0.1385 | Yes |
| 280 | BZW1 | basic leucine zipper and W2 domains 1 [Source:HGNC Symbol;Acc:HGNC:18380] | 11875 | -0.566 | -0.1356 | Yes |
| 281 | MICALL1 | MICAL like 1 [Source:HGNC Symbol;Acc:HGNC:29804] | 11912 | -0.574 | -0.1323 | Yes |
| 282 | NOP56 | NOP56 ribonucleoprotein [Source:HGNC Symbol;Acc:HGNC:15911] | 11939 | -0.580 | -0.1281 | Yes |
| 283 | PKP4 | plakophilin 4 [Source:HGNC Symbol;Acc:HGNC:9026] | 11942 | -0.581 | -0.1220 | Yes |
| 284 | CEMIP2 | cell migration inducing hyaluronidase 2 [Source:HGNC Symbol;Acc:HGNC:11869] | 11971 | -0.588 | -0.1179 | Yes |
| 285 | KTN1 | kinectin 1 [Source:HGNC Symbol;Acc:HGNC:6467] | 12003 | -0.593 | -0.1139 | Yes |
| 286 | RTN4 | reticulon 4 [Source:HGNC Symbol;Acc:HGNC:14085] | 12017 | -0.596 | -0.1085 | Yes |
| 287 | FER | FER tyrosine kinase [Source:HGNC Symbol;Acc:HGNC:3655] | 12101 | -0.620 | -0.1084 | Yes |
| 288 | EGFR | epidermal growth factor receptor [Source:HGNC Symbol;Acc:HGNC:3236] | 12109 | -0.622 | -0.1023 | Yes |
| 289 | YWHAE | tyrosine 3-monooxygenase/tryptophan 5-monooxygenase activation protein epsilon [Source:HGNC Symbol;Acc:HGNC:12851] | 12137 | -0.628 | -0.0976 | Yes |
| 290 | FASN | fatty acid synthase [Source:HGNC Symbol;Acc:HGNC:3594] | 12194 | -0.643 | -0.0952 | Yes |
| 291 | PKN2 | protein kinase N2 [Source:HGNC Symbol;Acc:HGNC:9406] | 12237 | -0.655 | -0.0914 | Yes |
| 292 | HNRNPK | heterogeneous nuclear ribonucleoprotein K [Source:HGNC Symbol;Acc:HGNC:5044] | 12341 | -0.688 | -0.0922 | Yes |
| 293 | PTPRH | protein tyrosine phosphatase receptor type H [Source:HGNC Symbol;Acc:HGNC:9672] | 12464 | -0.732 | -0.0940 | Yes |
| 294 | EEF2 | eukaryotic translation elongation factor 2 [Source:HGNC Symbol;Acc:HGNC:3214] | 12486 | -0.742 | -0.0876 | Yes |
| 295 | CAPZA1 | capping actin protein of muscle Z-line subunit alpha 1 [Source:HGNC Symbol;Acc:HGNC:1488] | 12512 | -0.751 | -0.0815 | Yes |
| 296 | LAD1 | ladinin 1 [Source:HGNC Symbol;Acc:HGNC:6472] | 12514 | -0.752 | -0.0735 | Yes |
| 297 | RPS2 | ribosomal protein S2 [Source:HGNC Symbol;Acc:HGNC:10404] | 12541 | -0.769 | -0.0673 | Yes |
| 298 | ITGB1 | integrin subunit beta 1 [Source:HGNC Symbol;Acc:HGNC:6153] | 12566 | -0.780 | -0.0608 | Yes |
| 299 | SLC3A2 | solute carrier family 3 member 2 [Source:HGNC Symbol;Acc:HGNC:11026] | 12587 | -0.789 | -0.0538 | Yes |
| 300 | RPL34 | ribosomal protein L34 [Source:HGNC Symbol;Acc:HGNC:10340] | 12602 | -0.799 | -0.0463 | Yes |
| 301 | ATXN2L | ataxin 2 like [Source:HGNC Symbol;Acc:HGNC:31326] | 12638 | -0.815 | -0.0403 | Yes |
| 302 | KIF5B | kinesin family member 5B [Source:HGNC Symbol;Acc:HGNC:6324] | 12662 | -0.835 | -0.0331 | Yes |
| 303 | LRRFIP1 | LRR binding FLII interacting protein 1 [Source:HGNC Symbol;Acc:HGNC:6702] | 12717 | -0.880 | -0.0279 | Yes |
| 304 | ARGLU1 | arginine and glutamate rich 1 [Source:HGNC Symbol;Acc:HGNC:25482] | 12732 | -0.895 | -0.0194 | Yes |
| 305 | RPL23A | ribosomal protein L23a [Source:HGNC Symbol;Acc:HGNC:10317] | 12785 | -0.944 | -0.0133 | Yes |
| 306 | DDX3X | DEAD-box helicase 3 X-linked [Source:HGNC Symbol;Acc:HGNC:2745] | 12788 | -0.949 | -0.0033 | Yes |
| 307 | CTNNB1 | catenin beta 1 [Source:HGNC Symbol;Acc:HGNC:2514] | 12866 | -1.119 | 0.0027 | Yes |
Table: GSEA details [plain text format]

  

Fig 2: GOMF\_CADHERIN\_BINDING      
 Blue-Pink O' Gram in the Space of the Analyzed GeneSet

  

Fig 3: GOMF\_CADHERIN\_BINDING: Random ES distribution      
 Gene set null distribution of ES for **GOMF\_CADHERIN\_BINDING**
